# The adult nasal mucosa is defined by distinct immune profiles that modulate in-vitro SARS-CoV-2 infection

**DOI:** 10.21203/rs.3.rs-8397474/v1

**Published:** 2026-01-29

**Authors:** Sarah N Gowanlock, Victor HK Lam, Zeynep Güneş Tepe, Daniel E Park, Vera Tai, Juan E Salazar, Tony Pham, Yazan Khan, Sydney Nelson, Caitlin Horn, David Zuanazzi, Diana Yang, Lance B Price, Rupert Kaul, Leigh Sowerby, Ryan M Troyer, Cindy M Liu, Jessica L Prodger

**Affiliations:** Western University; Western University; Western University; George Washington University; Western University; George Washington University; George Washington University; Western University; George Washington University; George Washington University; Western University; University of Toronto; George Washington University; University of Toronto; Western University; Western University; George Washington University; Western University

**Keywords:** Mucosal immunology, immune profiling, nasal mucosa, nasal inflammation, respiratory viruses, SARS-CoV-2, viral susceptibility

## Abstract

**Background:**

The nasal mucosa is the primary entry site for many respiratory viruses, and immune molecules present at the time of exposure may dictate if infection occurs. However, the baseline immune state in healthy adults – and how it influences susceptibility to viruses – remains poorly defined.

**Methods:**

Levels of 16 immune molecules were measured in nasal secretions from two independent cohorts of healthy adults (total n = 166, Luminex). Participants were clustered based on normalized concentrations of immune analytes to identify profiles. An *in vitro* organotypic model of the nasal epithelium was used to examine the effect of immune profiles on SARS-CoV-2 infection: primary human nasal epithelial cells (n = 9 donors) were grown at air-liquid interface to induce mucociliary differentiation (42 days), treated with recombinant human cytokines (72 hours), and then challenged with wildtype SARS-CoV-2 Omicron BA.1 (24 hours). SARS-CoV-2 entry factor expression (post-cytokines, pre-challenge) and viral infection (N gene) were measured by qRT-PCR.

**Results:**

In both cohorts, a unique cluster was observed, characterized by distinctly high levels of antiviral interferons – particularly IFN-λ3 – with comparatively low levels of inflammatory chemokines and cytokines. In contrast, individuals with high overall levels of inflammatory mediators had absent IFN-λ3. *In vitro*, pretreatment with IFN-λ3 and IFN-α2, but not with pro-inflammatory cytokines, significantly reduced SARS-CoV-2 replication in differentiated nasal epithelial cultures, despite upregulating ACE2 expression.

**Conclusions:**

Healthy adults exhibit distinct nasal immune profiles, with an IFN-λ3–dominant, low-inflammatory state conferring resistance to SARS-CoV-2 in vitro. The nasal immune milieu may influence susceptibility to respiratory viruses and the efficacy of mucosally administered vaccines.

## BACKGROUND

Respiratory viral infections represent a significant global public health challenge, contributing to substantial morbidity, mortality, and economic burden^1–3^. The COVID-19 pandemic, caused by the novel coronavirus SARS-CoV-2, served as a stark reminder of the persistent risks posed by respiratory viruses^4^. The nasal mucosa is the primary site for the attachment, entry, and initial replication of many respiratory viruses, including SARS-CoV-2^5–7^. Nasal epithelial cells express high levels of key viral entry factors, such as ACE2 and TMPRSS2, which facilitate SARS-CoV-2 binding and internalization^8–10^. However, nasal epithelial cells are also the first line of defence against infection, providing physical protection (direct barrier, mucus, and ciliary action), secreting antiviral factors (e.g., interferons), and coordinating the initial antiviral immune response (e.g., through production of chemokines and cytokines)^11^. Emerging evidence emphasizes the critical role that early interferon responses play in controlling viral replication and preventing viral progression to lower respiratory tract (LRT) infection^12,13^. In severe COVID-19 cases, delayed or suppressed type I and III IFN responses were frequently coupled with the activation of inflammatory mediators such as TNFα, MCP1, and MIP^14–16^. This dysregulated immune response contributes to the development of a hyperinflammatory state, or “cytokine storm”^15–18^, which is a hallmark of severe infection.

Despite the importance of the initial immune response to SARS-CoV-2 in determining disease severity, the factors determining early host responses are poorly understood, in part because it is difficult to study the events immediately preceding and during initial infection. Observational studies suggest that host factors (e.g., age, sex, genetics, microbiome and chronic illness), as well as environmental exposures (e.g., smoking), can alter the nasal mucosa and immune response to respiratory viral infections^19–22^. Children typically mount a more robust early innate immune response, characterized by elevated interferon signalling and cytokine production, whereas aging is associated with immunosenescence – a decline in immune cell function and response vigour – resulting in increased vulnerability to severe disease outcomes^23–25^. Smoking has been linked to impaired antiviral responses, such as reduced IFN-γ production upon influenza viral challenge^26,27^, caused by elevated baseline secretion of pro-inflammatory cytokines like IL8 and MCP1^28,29^. The local microbiome has been linked to differential immune responses to SARS-CoV-2, with variations in its composition modulating local IFN responses^30–32^. Pre-existing differences in nasal immunity may influence whether exposure to SARS-CoV-2 results in infection and, if infection occurs, disease severity^33,34^. However, whether these immune alterations translate into increased susceptibility to SARS-CoV-2 infection, or elevated viral burden and altered disease progression, is still not well understood. To date, most research has focused on immune responses during or after infection, leaving the baseline immune state of the nasal mucosa in healthy individuals poorly characterized.

To address this knowledge gap, we characterized the immune landscape of the nasal mucosa in two independent cohorts of healthy adults. We observed two distinct profiles of nasal immune molecules, reproduced across both cohorts. One profile was defined by high levels of type I and III interferons – IFN-α2 and IFN-λ3 – with low levels of prototypic proinflammatory cytokines. In contrast, a second profile comprised individuals with low IFN-λ3 levels but elevated inflammatory chemokines. To investigate the *ex vivo* functional impact of these distinct immune profiles, we pre-treated primary nasal epithelial cultures with representative immune mediators prior to challenge with SARS-CoV-2. We found that the specific interferon-associated nasal immune profile, but not pro-inflammatory nasal immune profiles, was protective against SARS-CoV-2 challenge.

## METHODS

### Study Participants and Sample Collection

This is a cross-sectional, retrospective study of samples collected from two observational cohorts. The Healthcare Worker (HCW) cohort was established for surveillance of COVID-19 seroconversion and asymptomatic infection among essential healthcare workers at George Washington University (GWU) in Washington, DC, United States, between 2020 and 2021. HCW participants volunteered to provide self-collected anterior nasal samples and completed questionnaires on demographic information, including sex, age, race, ethnicity, and medical history. The George Washington Occupational Health (GWOH) cohort participants comprised adults aged 18–45 who attended the GWOH Influenza Vaccination Clinic. GWOH participants provided self-collected anterior nasal swabs prior to vaccination and provided demographic information, including sex, age, race, ethnicity, medical history, and recreational tobacco history.

Samples included in this study were from 110 HCW participants and 79 GWOH participants who were negative for SARS-CoV-2 and without respiratory symptoms at the time of sampling. Among GWOH cohort participants, only pre-vaccination collected nasal swabs were used for this study. All participants provided written informed consent, and approval was obtained from the Human Subjects Research Ethics Board at Western University (project ID: 119682) and the Institutional Review Board at George Washington University (IRB# NCR202448 and NCR213697).

### Nasal swab collection

In the HCW and GWOH cohorts, participants self-collected anterior nasal swabs using 3D-printed lattice swabs (HCW, due to limited commercial availability of swabs early in the pandemic) or sterile nylon-flocked swabs (GWOH) into 500 μL of a stabilization buffer containing phosphate-buffered saline (PBS) with a protease inhibitor (cOmplete Mini Protease Inhibitor Cocktail, Roche), primocin antimicrobial agent (InvivoGen), and 10% bovine serum albumin (Sigma-Aldrich). Nasal swab eluent was aliquoted and stored at −80°C before transfer to Western University (London, Canada) for immune assays^35^.

### Immune analyte quantification

Sixteen immune analytes were chosen for quantification based on their previously described associations with inflammation, epithelial barrier function, and anti-viral defences: IFN-α2, IFN-λ3, IFN-γ, IL1α, IL1β, IL6, IL8/CXCL8, IL10, IL13, IL17A, TNFα, RANTES/CCL5, MIP1β/CCL9, MIG/CXCL9, IP10/CXCL10, and MUC5AC^36–49^. Of these analytes, IFNγ, IL10, IL13, IL17A, IL1β, IL6, IL8, MIP1β, and TNFα were quantified using the Luminex Human High Sensitivity T Cell Panel kit (HSTCMAG-28SK; Millipore Sigma); IFNα2, IL1α, IP10, MIG, and RANTES were quantified using the Luminex Human Cytokine/Chemokine/Growth Factor Panel A kit (HCYTA-60K; Millipore Sigma); IFN-λ3 was quantified using the Human Cytokine/Chemokine Magnetic Bead Panel IV (HCYP4MAG-64K; Millipore Sigma); and MUC5AC was measured by ELISA (EKN47139–96T; Biomatik). Millipore Multiplex Kits were used along with Luminex xMAP technology to detect immune molecules. Both Luminex and ELISA assays were performed according to the manufacturer’s instructions. Samples were run in duplicate, and Belysa immunoassay curve-fitting software (Belysa v1.2) was used to calculate coefficients of variation (CV), generate standard curves, and determine the lower limits of quantification (LLOQ). The LLOQ of each analyte in each cohort was assigned using Belysa Software based on the spread of unknowns (samples) relative to standards on the standard curve.

### Primary Nasal Epithelial Cultures

Mature nasal epithelial cultures with mucociliary differentiation were generated from primary cells isolated from participants undergoing skull base surgery in London, Ontario. Isolation of primary nasal epithelial cells, generation of mature nasal epithelial cultures, tissue processing, and imaging were performed according to the protocol published by Lam et al.^50^. Detailed methods are also provided in the Supplementary Information. All tissue donors provided written informed consent, and approval was obtained from the Human Subjects Research Ethics Board at Western University (project ID: 119682).

### SARS-CoV-2 challenge

Mature nasal epithelial cultures (42 days post-ALI) were used for SARS-CoV-2 challenge. The apical mucus was removed by washing the apical surface of the inserts with 100 μl PBS (Wisent Inc.). Then, the basolateral medium was replaced with PneumaCult-ALI medium supplemented with a reduced hydrocortisone concentration (200 nM) and cytokine cocktails representing physiologically relevant concentrations of immune proteins comprising the chemotactic and IFN-high immune profiles. The chemotactic IPT cocktail included MIP-1β (200 ng/mL; 271-BME-010/CF, R&D Systems), RANTES (200 ng/mL; 278-RN-010/CF, R&D Systems), TNF-α (50 ng/mL; 210-TA-020/CF, R&D Systems), IP-10 (400 ng/mL; 266-IP-010/CF, R&D Systems), IL-8 (200 ng/mL; 208-IL-010/CF, R&D Systems), and MIG (400 ng/mL; 392-MG-010/CF, R&D Systems). The IFN-high IPT cocktail consisted of IFN-λ3 (1 ng/mL; 5259-IL-025/CF, R&D Systems) and IFN-α2 (5 ng/mL; NBP2–34971, Novus Biologicals). All procedures using infectious SARS-CoV-2 were performed in a BSL-3 facility at the Imaging Pathogens for Knowledge Translation (ImPaKT) facility following standard safety guidelines. Optimal SARS-CoV-2 infection conditions were assessed across various multiplicities of infection (MOI: 0.001, 0.01, 0.1) and durations of infection (24-, 48- and 72-hours post-infection (hpi)). Optimization data is presented in **Supplementary Fig. 1**. Cells were pre-treated with immune mediators for 72 hours prior to SARS-CoV-2 challenge. Fresh cytokines and media (reduced hydrocortisone) were replenished 24 hours prior to challenge. Infection with SARS-CoV-2 Omicron BA.1 (from the British Columbia Centre for Disease Control Public Health Laboratory) was conducted at an MOI of 0.01 by adding 100 μl viral inoculum to the apical side of cells and incubating for 1 hour at 37°C. After infection, the viral inoculum was removed, the cells were washed three times with 100 μl PBS and then incubated at 37°C. Twenty-four hours after viral challenge, apical surfaces of cells were washed three times with 100 μl PBS to remove secreted virus. Cells were then lysed with 200 μl TRIzol reagent (Invitrogen) and incubated at room temperature for 10 min. RNA was extracted using the PureLink RNA Mini Kit (Invitrogen), and intracellular viral load was measured via qRT-PCR for SARS-CoV-2 N gene as described previously^50^. Corrected cycle threshold (Ct) values were calculated by subtracting the Ct of the gene of interest from that of the housekeeping gene, ribosomal protein lateral stalk subunit P0 (RPLP0). Infections were performed on mature epithelia generated from n = 9 donors, in quadruplicate.

### SARS-CoV-2 receptor mRNA quantification

Mature nasal epithelial cultures (42 days post-ALI) were used to assess the impact of cytokine cocktails on SARS-CoV-2 receptor expression. As described above, apical mucus was removed, and basolateral media were replaced with PneumaCult-ALI medium containing a reduced concentration of hydrocortisone (200 nM) and IPT cytokine cocktails. After 72 hours, with replenishment of fresh cytokines and medium at 48 hours, cells were lysed and collected with TRIzol reagent, and then RNA was extracted using the PureLink RNA Mini Kit (InVitrogen). SARS-CoV-2 receptor expression (ACE2 and TMPRSS2) was quantified by qRT-PCR, as previously described^50^. This experiment was performed in triplicate using tissues from three different donors.

### Statistical analysis

#### In vivo nasal immune characterization

All analyses were performed in R version 4.3.1. Samples with an analyte concentration above the LLOQ but with a CV > 20% were re-run once, and if the CV remained high, the datapoint was excluded from analyses. Detection of IL1α, which is constitutively expressed in the nasal mucosa^51^, was used as a marker of adequate self-sampling. Of the 110 self-collected HCW samples, 23 were excluded based on undetectable IL-1α, leaving n = 87 participants; all GWOH samples had detectable IL1α.

Due to differences in sample collection protocols, analyses were performed independently for each cohort unless otherwise indicated. Data was analyzed using R (version 4.4.2) and RStudio (2023.06.1 + 524) with the following packages: cluster (v2.1.6), dplyr (v1.1.4), factoextra (v1.0.7), ggplot2 (v3.5.1), ggpubr (v0.6.0), indicspecies (v1.8.0), reshape2 (v1.4.4), rstatix (v0.7.2), stringr (v1.5.1), tidyverse (v2.0.0), and vegan (v2.6–10).

Analyte concentrations were log_10_-transformed and converted to percentiles relative to the highest measured value for each analyte; a pseudo-value of 1 was added to all concentrations to prevent taking the log of zeros. Immune analyte clustering was performed independently for each cohort using the k-medoids clustering algorithm (PAM) on a Bray-Curtis distance matrix, with k set to five clusters. The number of clusters was selected using a traditional elbow method with the function ‘fviz_nbclust’ from the *factoextra* package in R (parameters: method=“wss”, FUNcluster = pam, nboot = 500).

For each cohort, immune analyte concentrations (log_10_-transformed pg/ml) were compared between clusters using Kruskal-Wallis tests, followed by pairwise Dunn tests with false discovery rate (FDR) adjustment. Within each cohort, multilevel pattern analyses [indicspecies package function ‘multipatt’ (parameters: func=“IndVal.g”, duleg = TRUE, permutations = 999)] were used to identify analytes that significantly contributed to the clustering pattern. The relative abundances of immune analytes were compared between participants with a high-IFN immune profile vs. all other participants using the Wilcoxon rank-sum test with Benjamini-Hochberg FDR correction.

Demographic variables were analyzed separately for each cohort. Kendall’s rank correlation test was used for age, and a Wilcoxon rank-sum test was applied to biological sex and racial groups. The Fisher’s Exact Test was used for categorical variables. The log_10_-transformed concentrations of each analyte were compared across categorical groups using the Wilcoxon rank-sum test (for two-group comparisons) or the Kruskal-Wallis rank sum test (for variables with more than two categories), followed by a Dunn test with FDR adjustment when appropriate. Associations between analyte concentration and continuous demographic variables were assessed using the Kendall’s rank correlation test.

#### Ex vivo mature airway epithelia experiments

To assess differences in both SARS-CoV-2 receptor expression and SARS-CoV-2 replication, a row-matched one-way ANOVA was performed across IPT pre-treatment groups. Post hoc comparisons between each IPT group and the unstimulated control were conducted using an uncorrected Fisher’s Least Significant Difference (LSD) test.

## RESULTS

### The adult nasal mucosa is host to distinct nasal immune profiles

The HCW analytic cohort (n = 87) consisted primarily of young adults (median age 32 years, IQR 30–42.8) with a balanced sex distribution (male 55%, female 45%). Participant characteristics are summarized in Table 1.

**Figure 1:**
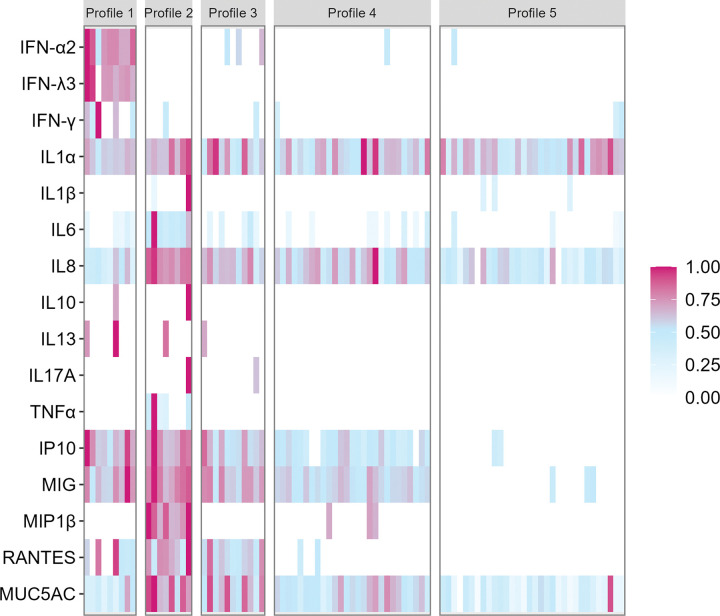
Adults form five distinct clusters based on secreted immune mediators. Concentrations of immune analytes were quantified in nasal swabs self-collected by 87 participants from the HCW cohort. Each vertical column represents one sample from a single participant, and each square represents the relative concentration (normalized to the highest observed concentration in the study) of the specific immune analyte. Participants were clustered based on their relative concentration of all immune analytes (partition around medoids (PAM), k=5).

We identified five distinct nasal immune profiles among the participants using a clustering analysis of the panel of normalized concentrations (relative to the maximum) of immune analytes (Fig. 1). Nasal immune profile clusters 1 and 2 appeared distinct from the remaining clusters. Cluster 1 was defined by significantly higher relative concentrations of interferons, including IFN-λ3, IFN-α2, and IFN-γ (all p < 0.01), while individuals in cluster 2 were defined by significantly higher inflammatory markers TNF-α, IL6, RANTES, IP10, MIG, MUC5AC, IL8, IL1β, and MIP-1β (all p < 0.05). Participants in clusters 3 to 5 had successively lower levels of inflammatory chemokines and cytokines, including IL8, IP10, MIG, RANTES, and MUC5AC. Detailed pairwise comparisons across nasal immune profile clusters can be found in **Supplemental Table 1**.

### Validation of Nasal Immune Profiles in an Independent Cohort

Nasal swabs were collected from a second cohort of 79 participants attending a flu vaccine clinic (GWOH) at GWU in 2022 and analyzed to validate the observed nasal immune profiles. Participants from this GWOH cohort were younger (median age 19 vs. 32 years, p < 0.001; IQR 18.0–23.0), more likely to be white (70% vs 41%, p < 0.01), and less likely to be black (or of African American descent) than the HCW cohort (6% vs 28%, p < 0.01; Table 2). However, they had a similar balanced sex distribution (male 52%, female 48%). Demographic data from the GWOH cohort are presented in Table 2.

**Figure 2:**
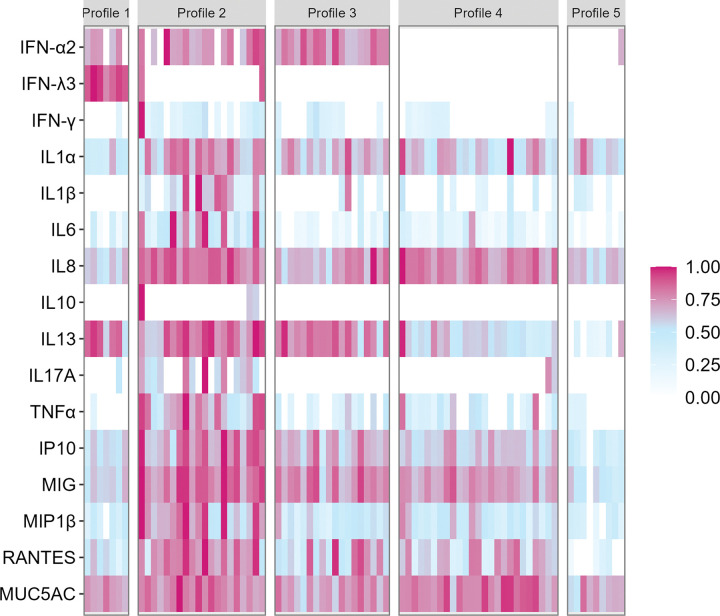
GWOH cohort participants form five distinct clusters based on secreted immune mediators. Concentrations of immune analytes were quantified in nasal swabs self-collected by 79 adults in the GWOHcohort. Each vertical column represents one participant, and each square represents the relative abundance of the indicated immune analyte (proportional to the highest observed concentration). Participants were clustered based on their relative abundances of immune analytes (partition around medoids (PAM), k=5).

In general, immune analyte concentrations were higher in the GWOH cohort compared to the HCW cohort (**Supplemental Table 2**), and additional demographic data were collected. Nevertheless, as in the HCW cohort, this analysis revealed five distinct clusters of participants with a strong dichotomy between GWOH cluster 1, similar to the previously identified HCW cluster 1, and the other clusters; in the GWOH cohort, however, this dichotomy was driven solely by IFN-λ3 (Fig. 2). Similar to the HCW cohort, immune analyte concentrations sequentially decreased from cluster 2 to 5, with participants in cluster 5 having overall low levels of all immune analytes. A full list of the significant pairwise differences in soluble immune molecules across GWOH clusters is provided in **Supplementary Table 3**. After accounting for false discovery, no significant associations were observed between any participant demographics and immune cluster membership.

### Association between nasal immune markers and host characteristics

Host demographics were associated with specific nasal immune markers in the GWOH cohort. Female sex was associated with significantly lower IL1α (median 1.75 vs. 2.09 log_10_ (value + 1) pg/ml, p < 0.01) and IL1β (median 0.00 vs 0.34 log_10_ (value + 1) pg/ml, p < 0.01) than male sex (**Supplemental Fig. 2**). Age was negatively correlated with relative concentrations of RANTES, IL6, IP10, and MIG (all p < 0.05; **Supplemental Fig. 3**). Compared to white participants, Asian study participants had significantly lower levels of pro-inflammatory cytokines and chemokines, including MIG, IL-6, TNF-α, IP10, MIP1β, and IL-8 (all p < 0.05; **Supplemental Fig. 4**). Having a history of seasonal allergies was associated with significantly higher levels of IFN-γ, IL17A, IL6, and MIP1β (all p < 0.05), while a history of asthma was associated with higher levels of TNFα (p < 0.05; both **Supplemental Fig. 5**). While female sex was statistically associated with higher IL13 concentrations in the HCW cohort (Wilcoxon rank-sum test: W = 1032, p < 0.05), IL13 was detected in only 3% of individuals. No other associations between host demographics and specific immune markers were observed in the HCW cohort.

While smoking was relatively uncommon among GWOH cohort participants, some patterns emerged with specific nasal immune markers. Participants who reported having ever using a hookah had significantly lower concentrations of IL1α and IL6 compared to those who reported never having used (IL1α median 1.61 vs 1.96 and IL6 median 0.30 vs 0.61 log_10_ (value + 1) pg/ml, both p < 0.05), participants reporting regular use of e-cigarettes (used on > 14 of the past 30 days), were more likely to have detectable IFN-λ3 compared to those who did not regularly use e-cigarettes (2/2 vs 7/65, p < 0.05), and participants who had ever smoked a cigar had higher levels of IL13 compared to those who had not (median 1.23 vs 0.97 log_10_ (value + 1) pg/ml, p < 0.05; **Supplemental Fig. 6**).

### Pretreatment with type I and III interferons, but not chemokines, reduces the susceptibility of primary nasal epithelial culture to SARS-CoV-2 infection

**Figure 3:**
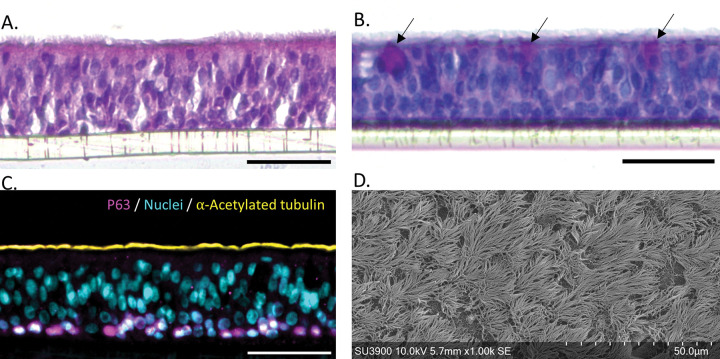
Characterization of mature nasal epithelial tissues. **(A)** Hematoxylin and eosin (H&E) staining demonstrating the pseudostratified organization of nasal epithelia. **(B)** Periodic acid–Schiff (PAS) staining highlights mucus-producing goblet cells, with black arrows indicating glycogen-rich mucus residues on the nasal epithelium. **(C)** Immunofluorescent staining revealing tissue composition: acetylated α-tubulin (cilia, yellow), p63 (basal/proliferative cells, magenta), and DAPI (nuclei, cyan). **(D)** Scanning electron microscopy (SEM) image showing cilia formation. Scale bars: 50 μm. Brightness and contrast were adjusted to improve visualization.

To assess the functional impact of two highly contrasting nasal immune profiles on modifying nasal mucosal susceptibility to respiratory viral infection, we tested the indicator immune markers from nasal immune profile clusters 1 and 2 – high interferons vs. high pro-inflammatory cytokines – in a primary nasal epithelial culture. Representative images of mature nasal epithelial cultures are shown in Fig. 3, illustrating pseudostratified epithelium (Fig. 3A) with mucociliary differentiation, including goblet cell formation (Fig. 3B) and cilia (Fig. 3C–D).

**Figure 4:**
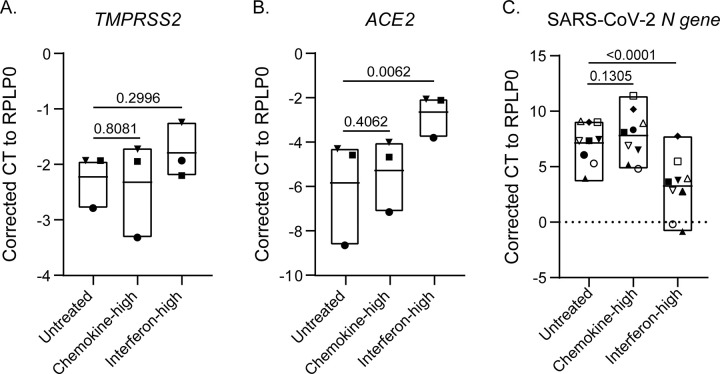
Interferons, but not other inflammatory mediators, protect mature nasal airway cultures from SARS-CoV-2, despite increasing ACE2 gene expression. Mature organotypic nasal epithelial cultures were stimulated with cytokine cocktails representative of IFN-high or chemokine-high immune profiles for 72 hours prior to SARS-CoV-2 infection. Prior to infection, cells were harvested to quantify **(A)** TMPRSS2 and **(B)** ACE2 transcript levels by RT-qPCR, normalized to RPLP0 (n=3 donors). **(C)** The IFN-high profile significantly reduced SARS-CoV-2 N gene expression compared to unstimulated controls, while the chemokine-high profile had no significant effect (n=9 donors). Each data point represents the mean of either triplicate or quadruplicate wells from an individual donor; data point symbols indicate tissue donors. Statistical analysis was performed using a row-matched one-way ANOVA, followed by post hoc comparisons to the unstimulated control using an uncorrected Fisher’s LSD test. p-values are indicated on the graphs.

We first assessed how exposure to the indicator immune markers from clusters 1 and 2 affects expression of the SARS-CoV-2 receptor (ACE2) and co-factor (TMPRSS2) in primary nasal epithelial cultures (Fig. 4A–B). While neither treatment significantly impacted TMPRSS2 expression, as compared to unstimulated controls (ΔCt: controls = −1.792 vs. cluster 1 = −2.321 vs. cluster 2- = −2.226), epithelial cultures treated with cluster 1 (interferon-high) profile had significantly increased ACE2 expression compared to unstimulated controls (ΔCt: −2.648 vs. −5.837, p = 0.006) (Fig. 4B).

We further tested how treatment with recombinant human protein mixtures, broadly recapitulating the two immune profiles of interest, impact nasal epithelial cultures susceptibility to replication-competent SARS-CoV-2 Omicron Ba.1. This showed that epithelial cultures treated with cluster 1 (interferon-high) profile had significantly lower mean SARS-CoV-2 N gene expression after 24 hours than unstimulated controls (ΔCt: control = 7.14 vs. cluster 1 = 3.26) Fig. 4C). In contrast, treatment with the cluster 2 (chemokine-high) profile had similar SARS-CoV-2 N gene expression to unstimulated controls (ΔCt: control = 7.14 vs. cluster 2 = 7.81; p = 0.1305, Fig. 4C).

## DISCUSSION

The nasal mucosa is the primary site of viral entry and plays a critical role in shaping the course of viral infections and their management. In this study, we identified two distinct, reproducible nasal immune profile types in healthy adults: a high-interferon (IFN-λ3–dominant) low-inflammatory state and a pro-inflammatory/chemotactic low-interferon state. This dichotomy was consistently observed across two independent cohorts, despite differences in swab collection, participant demographics, and time, underscoring their robustness. We found that the high-interferon profile, but not the high pro-inflammatory/chemotactic profile, protected against wildtype SARS-CoV-2 in vitro, suggesting the baseline immune status of the nasal mucosa is a critical determinant of SARS-CoV-2 susceptibility.

We assessed the functional role of immune profiles using differentiated primary human nasal epithelial cultures. In vitro pretreatment of nasal epithelia with interferons (IFN-λ3 and IFN-α2) but not pro-inflammatory cytokines (MIP-1β, RANTES, TNF-α, IP-10, IL-8, MIG) conferred significant protection against SARS-CoV-2 Omicron BA.1 challenge. These findings suggest that a naturally occurring IFN-λ3–dominant, low-inflammatory immune state in the nasal mucosa may confer intrinsic resistance to infection, consistent with the ability of IFN-λ to promote epithelial antiviral programs while restraining chemokine-driven inflammation^52–55^. The antiviral protection we observed is likely mediated by robust induction of interferon-stimulated genes (ISGs) in epithelial cells, conferring a cell-intrinsic antiviral state. We confirmed the well-established link between IFN signalling and ACE2 expression: IFN pretreatment upregulated ACE2 in our primary nasal epithelial model. This observation not only supports the known role of IFNs in modulating ACE2 expression but also validates the relevance of our in vitro model to in vivo conditions, where similar ACE2 upregulation occurs in response to IFN signaling^56–58^. Despite the increase in ACE2, infection levels still decreased, indicating that the antiviral effects driven by IFN responses override any potential enhancement of viral entry due to increased ACE2 expression. This finding helps reconcile conflicting reports on ACE2 modulation and viral susceptibility^57,59^. Our data therefore reinforce the complex interplay between viral entry factors and innate immune defences in primary human tissues, demonstrating the multifaceted role of cytokines during viral infection.

Our results extend prior murine and in vitro studies showing that IFN-λ exerts potent antiviral activity without inducing systemic inflammatory responses^60–64^. Unlike type I or II IFNs, IFN-λ receptors are predominantly expressed on epithelial cells, limiting their activity to mucosal surfaces^65,66^. In respiratory viral infection models, IFN-λ limits neutrophil recruitment and IL1-driven pathology, preserving tissue integrity while enhancing local antiviral defence^52,54^. Importantly, most previous work has examined experimentally induced IFN-λ responses^53,67,68^, whereas our study demonstrates that this protective, low-inflammatory immune tone exists naturally in a subset of healthy adults. This supports recent models, proposing that baseline IFN-λ activity sets a mucosal “antiviral tone” that can influence infection outcomes. This phenomenon may also explain why certain individuals remain asymptomatic and quickly clear the virus, while others are more prone to developing severe disease.

Despite several associations between individual immune analytes and participant demographics or airway exposures, none correlated with immune cluster membership. Age was negatively associated with multiple analytes, consistent with immunosenescence shaped by lifelong antigen exposure and environmental stressors^69,70^. Asthmatic participants had elevated TNFα, in line with its role in airway inflammation^71^, and those with seasonal allergies showed higher IFN-γ, IL17A, IL6, and MIP-1β, reflecting the mixed Th1/Th17 response typical of allergic airway disease^72–75^. Female participants had lower nasal IL1α/β than males, consistent with evidence that sex hormones modulate IL1β secretion and that men mount stronger IL1 responses^76–78^. Asian participants had lower levels of several cytokines, paralleling reports of divergent T-effector polarization in chronic sinus disease^79^. Tobacco exposures also shaped responses: environmental smoke is linked to Th2 skewing^80^, matching our finding that cigar users had higher IL13. Notably, cigarette smoke and nicotine impair production of type I/II IFN in response to the live-attenuated influenza vaccination^81–83^. We observed that frequent e-cigarette use was associated with higher baseline IFN-λ3, which protected against SARS-CoV-2 in vitro. If high IFN-λ3 similarly inhibits the replication of live-attenuated vaccines, this could dampen vaccine-induced immune priming. Further research on the effect of the pre-existing immune milieu on variability in mucosal vaccine efficacy is warranted^84,85^. While informative for hypothesis generation, our cross-sectional design precludes conclusions on confounding, directionality, or causality.

A key unresolved question is what factors drive the emergence of these distinct nasal immune profiles in healthy adults. The nasal microbiome is a strong candidate, as commensal bacteria can modulate epithelial signalling at mucosal surfaces, influencing basal immune tone and antiviral readiness^86–89^. Variability in microbial community structure could therefore prime the mucosa toward either an IFN-λ–dominant antiviral state or a more chemotactic/pro-inflammatory state. Host intrinsic factors, including genetic variations in immune-related genes and signalling pathways, can further shape protein expression profiles and, in turn, contribute to inter-individual differences in steady-state mucosal immunity^90–94^. Environmental exposures, such as allergens^95–97^, air pollutants^98–100^, and tobacco smoke^101,102^, also recalibrate mucosal immune responses, suppressing or skewing cytokine output. Longitudinal and multi-omics approaches integrating microbiome composition, host genetics, and exposure histories will be essential to define the drivers and stability of these immune profiles over time.

Our findings have translational relevance, as IFN-λ appears to balance two essential functions of the mucosal barrier – providing robust antiviral defence while limiting collateral inflammation – thereby enabling viral control without excessive tissue injury. Baseline IFN-λ3 levels in the nasal mucosa could serve as a biomarker for mucosal resistance, supporting assessment of host defence capacity or informing targeted prophylactic approaches. Intranasal IFN-λ delivery has shown promise in pre-exposure prophylaxis models, including reduced SARS-CoV-2 infection and transmission in rodents^62,67,103^. It could be further explored in humans to transiently boost epithelial antiviral tone. However, the benefits of IFN-λ must be weighed against its potential to delay epithelial repair during prolonged signalling^54,104^, underscoring the need for careful optimization of dosing and timing.

## CONCLUSION

This study provides valuable insights into patterns of expression of nasal immune mediators and their impact on susceptibility to SARS-CoV-2 infection. Our findings demonstrate that a naturally occurring IFN-λ3–dominant, low-inflammatory nasal immune profile exists in healthy adults, and suggest this profile confers protection against SARS-CoV-2. These findings support a model in which IFN-λ acts as a central determinant of mucosal immune tone, promoting robust epithelial antiviral responses while limiting inflammation-driven pathology. Understanding the determinants and stability of this set-point may help identify individuals at increased risk for severe respiratory viral disease and guide the development of targeted prophylactic strategies.

### Limitations of the study

This study has several limitations. First, its observational cross-sectional design does not allow for causal inference regarding the relationships between baseline immune analytes and participant characteristics. While reproducible immune profiles were identified across two independent cohorts, direct cross-cohort comparisons were limited by methodological differences. In particular, the HCW cohort relied on 3D-printed swabs during early pandemic shortages, whereas the GWOH cohort used standardized commercial swabs under direct observation at a later stage of the pandemic, when participants were more familiar with self-collection. These differences likely contributed to the generally higher analyte concentrations observed in the GWOH cohort and complicate direct comparisons^105^.

While pretreatment with chemokines did not alter susceptibility in vitro, our ex vivo model lacked immune cells, restricting assessment to direct effects of soluble mediators on epithelial infection. Inflammatory chemokines may exert important effects in vivo by recruiting and activating immune cells^106^, functions not captured by our model. Additionally, severe SARS-CoV-2 disease is characterized by a hyperinflammatory response with elevated serum levels of IL1β, IL1Rα, IL6, IL7, IL10, IP10, and TNF-α, alongside an impaired IFN response^15,17,106^, mirroring the chemokine-high nasal profile observed in our participants. Individuals with a chemokine-high, interferon-low profile may be more vulnerable to severe COVID-19 pathogenesis, but this could not be explored in our ex vivo model. Expanded co-culture models incorporating immune cells will be required to dissect these interactions in a multicellular context. Understanding these dynamics could be critical for identifying individuals at higher risk and developing strategies to mitigate severe disease.

Finally, our sample size was modest and limited to adults up to 72 years of age. The generalizability of these findings to pediatric or older populations, or to other respiratory viruses, remains to be determined.

## Supplementary Material

Supplementary Files

This is a list of supplementary files associated with this preprint. Click to download.

• SourceData.xlsx

• SupplementalFile1.docx

## Figures and Tables

**Table 1:** Healthcare Worker (HCW) cohort participant characteristics

	HCW (n = 87)
**Age, years**	30.0–42.8
Age, median (Q1-Q3)	
**Sex**, n, %	
*Female*	39 (45%)
*Male*	48 (55%)
**Race**	
*White*	36 (41%)
*Black or African American*	24 (28%)
*Asian*	22 (25%)
*American Indian or Alaska Native*	1 (1.1%)
*More than one race*	4 (4.6%)
**Medical History**	
*Lung Disease*	11 (13%)
*Heart Disease*	2 (2.3%)
*Diabetes*	3 (3.4%)
*Cancer*	2 (2.3%)

**Table 2:** Demographics of participants in the George Washington Occupational Health (GWOH) cohort.

	n = 79
**Age** (median, in years)	19
**Biological sex**, n, %	
*Female*	38 (48%)
*Other*	1 (1%)
**Race**	
*White*	55 (70%)
*Black or African American*	5 (6.3%)
*Asian*	13 (16%)
*American Indian or Alaska Native*	0 (0%)
*More than one race*	6 (7.6%)
**Medical History**	
*Asthma*	11 (14%)
*Allergies*	36 (46%)
**Children residing in household**	4 (5.1%)
**Pets residing in household**	10 (13%)
**Antibiotic use (last 14 days)**	8 (10%)
**People residing in household**	**n = 69 respondents**
*1*	7 (10%)
*2*	27 (39%)
*3*	6 (9%)
*4*	29 (42%)
**Lifetime inhaled nicotine use**	**n = 69 respondents**
*Any*	32 (48%)
*Cigarette*	25 (36%)
*Electronic cigarette/vape*	18 (26%)
*Cigar*	15 (22%)
*Hookah*	13 (19%)
**Cumulative lifetime cigarette use**	**among 25 who used**
*1 cigarette*	5 (16%)
*2–5 cigarettes*	14 (56%)
*6–15 cigarettes*	3 (12%)
*15–25 cigarettes*	0
*26 + cigarettes*	3 (12%)

## Data Availability

The datasets supporting the conclusions of this article are included within the article (and its additional files).

## References

[1] Zimmerman RK, Balasubramani GK, D’Agostino HEA, Clarke L, Yassin M, Middleton DB, Population-based hospitalization burden estimates for respiratory viruses, 2015–2019. Influenza Other Respir Viruses. 2022;16:1133. 35996836 10.1111/irv.13040 PMC9530548

[2] Jin X, Ren J, Li R, Gao Y, Zhang H, Li J Global burden of upper respiratory infections in 204 countries and territories, from 1990 to 2019. EClinicalMedicine 2021; 37. 10.1016/j.eclinm.2021.100986 PMC8343248 34386754

[3] Stockwell MS, Reed C, Vargas CY, Wang L, Alba LR, Jia H, Five-Year Community Surveillance Study for Acute Respiratory Infections Using Text Messaging: Findings From the MoSAIC Study. Clin Infect Dis. 2022;75:987–95. 35037056 10.1093/cid/ciac027 PMC9383201

[4] Li J, Lai S, Gao GF, Shi W. The emergence, genomic diversity and global spread of SARS-CoV-2. Nature 2021 600:7889 2021; 600: 408–418. 10.1038/s41586-021-04188-6 34880490

[5] Pilapitiya D, Wheatley AK, Tan HX. Mucosal vaccines for SARS-CoV-2: triumph of hope over experience. EBioMedicine. 2023;92:104585. 37146404 10.1016/j.ebiom.2023.104585 PMC10154910

[6] Russell MW, Mestecky J. Mucosal immunity: The missing link in comprehending SARS-CoV-2 infection and transmission. Front Immunol. 2022;13:957107. 36059541 10.3389/fimmu.2022.957107 PMC9428579

[7] Vareille M, Kieninger E, Edwards MR, Regamey N. The Airway Epithelium: Soldier in the Fight against Respiratory Viruses. Clin Microbiol Rev. 2011;24:210. 21233513 10.1128/CMR.00014-10 PMC3021210

[8] Yang C, Li Y, Xiao S-Y. Differential expression of ACE2 in the respiratory tracts and its relationship to COVID-19 pathogenesis. 2020. 10.1016/j.ebiom.2020.102976 PMC7511839 32979833

[9] Jackson CB, Farzan M, Chen B, Choe H. Mechanisms of SARS-CoV-2 entry into cells. Nat Rev. 2022. 10.1038/s41580-021-00418-x. PMC8491763 34611326

[10] Ziegler CGK, Allon SJ, Nyquist SK, Mbano IM, Miao VN, Tzouanas CN, SARS-CoV-2 Receptor ACE2 Is an Interferon-Stimulated Gene in Human Airway Epithelial Cells and Is Detected in Specific Cell Subsets across Tissues. Cell. 2020;181:1016. 32413319 10.1016/j.cell.2020.04.035 PMC7252096

[11] Gallo O, Locatello LG, Mazzoni A, Novelli L, Annunziato F. The central role of the nasal microenvironment in the transmission, modulation, and clinical progression of SARS-CoV-2 infection. Mucosal Immunol. 2021;14:305. 33244161 10.1038/s41385-020-00359-2 PMC7690066

[12] Hatton CF, Botting RA, Dueñas ME, Haq IJ, Verdon B, Thompson BJ Delayed induction of type I and III interferons mediates nasal epithelial cell permissiveness to SARS-CoV-2. Nature Communications 2021 12:1 2021; 12: 1–17. 10.1038/s41467-021-27318-0 PMC8651658 34876592

[13] McNab F, Mayer-Barber K, Sher A, Wack A, O’Garra A. Type I interferons in infectious disease. Nature Reviews Immunology 2015 15:2 2015; 15: 87–103. 10.1038/nri3787 PMC7162685 25614319

[14] Savan R, Gale M. Innate immunity and interferon in SARS-CoV-2 infection outcome. Immunity. 2023;56:1443–50. 37437537 10.1016/j.immuni.2023.06.018 PMC10361255

[15] Eskandarian Boroujeni M, Sekrecka A, Antonczyk A, Hassani S, Sekrecki M, Nowicka H, Dysregulated Interferon Response and Immune Hyperactivation in Severe COVID-19: Targeting STATs as a Novel Therapeutic Strategy. Front Immunol. 2022;13:888897. 35663932 10.3389/fimmu.2022.888897 PMC9156796

[16] Blanco-Melo D, Nilsson-Payant BE, Liu WC, Uhl S, Hoagland D, Møller R, Imbalanced Host Response to SARS-CoV-2 Drives Development of COVID-19. Cell. 2020;181:1036–e10459. 32416070 10.1016/j.cell.2020.04.026 PMC7227586

[17] Tan LY, Komarasamy TV, Balasubramaniam RMT. Hyperinflammatory Immune Response and COVID-19: A Double Edged Sword. Front Immunol. 2021;12:742941. 34659238 10.3389/fimmu.2021.742941 PMC8515020

[18] Lee AJ, Ashkar AA. The Dual Nature of Type I and Type II Interferons. Front Immunol. 2018;9:2061. 30254639 10.3389/fimmu.2018.02061 PMC6141705

[19] Wang B, Li R, Lu Z, Huang Y. Does comorbidity increase the risk of patients with COVID-19: evidence from meta-analysis. Aging. 2020;12:6049. 32267833 10.18632/aging.103000 PMC7185114

[20] Wu Z, McGoogan JM. Characteristics of and Important Lessons From the Coronavirus Disease 2019 (COVID-19) Outbreak in China: Summary of a Report of 72 314 Cases From the Chinese Center for Disease Control and Prevention. JAMA. 2020;323:1239–42. 32091533 10.1001/jama.2020.2648

[21] Ramasamy S, Subbian S. Critical determinants of cytokine storm and type i interferon response in COVID-19 pathogenesis. Clin Microbiol Rev. 2021;34. 10.1128/CMR.00299-20/.ASSET/DAC9DCC4-328D-492F-A7D0-CD80616C5C51/ASSETS/IMAGES/LARGE/CMR.00299-20-F0003.JPG. PMC8142516 33980688

[22] Brazer N, Servellita V, Jin C, Foresythe A, Oseguera M, Nguyen J, Differential severity of SARS-CoV-2 variant infections in children and adults with COVID-19. J Clin Virol. 2025;180. 10.1016/j.jcv.2025.105833. 40674918

[23] Carsetti R, Quintarelli C, Quinti I, Piano Mortari E, Zumla A, Ippolito G, The immune system of children: the key to understanding SARS-CoV-2 susceptibility? Lancet Child Adolesc Health. 2020;4:414–6. 32458804 10.1016/S2352-4642(20)30135-8 PMC7202830

[24] Pierce CA, Sy S, Galen B, Goldstein DY, Orner E, Keller MJ, Natural mucosal barriers and COVID-19 in children. JCI Insight. 2021;6. 10.1172/JCI.INSIGHT.148694. PMC8262299 33822777

[25] Winkley K, Banerjee D, Bradley T, Koseva B, Cheung WA, Selvarangan R Immune cell residency in the nasal mucosa may partially explain respiratory disease severity across the age range. Scientific Reports 2021 11:1 2021; 11: 1–9. 10.1038/s41598-021-95532-3 PMC8342554 34354210

[26] Vlasma JR, van der Veen TA, De Jager MH, Nawijn MC, Brandsma C-A, Melgert BN. Cigarette smoking prolongs inflammation associated with influenza infection and delays its clearance in mice. Am J Physiology-Lung Cell Mol Physiol. 2024;327. 10.1152/AJPLUNG.00369.2023. 39254089

[27] Noah TL, Zhou H, Monaco J, Horvath K, Herbst M, Jaspers I. Tobacco Smoke Exposure and Altered Nasal Responses to Live Attenuated Influenza Virus. Environ Health Perspect. 2010;119:78. 20920950 10.1289/ehp.1002258 PMC3018504

[28] Jaspers I. Cigarette smoke effects on innate immune mechanisms in the nasal mucosa: Potential effects on the microbiome. Ann Am Thorac Soc. 2014;11. 10.1513/ANNALSATS.201306-154MG/SUPPL_FILE/DISCLOSURES.PDF$AUTHORDISCLOSURES. 24437404

[29] Lee J, Taneja V, Vassallo R. Cigarette Smoking and Inflammation: Cellular and Molecular Mechanisms. J Dent Res. 2012;91:142. 21876032 10.1177/0022034511421200 PMC3261116

[30] Park DE, Aziz M, Salazar JE, Pham T, Nelson SG, Villani J, The nasal microbiome modulates risk for SARS-CoV-2 infection. EBioMedicine. 2025;115. 10.1016/j.ebiom.2025.105660. PMC12143206 40210576

[31] Kolhe R, Sahajpal NS, Vyavahare S, Dhanani AS, Adusumilli S, Ananth S, Alteration in nasopharyngeal microbiota profile in aged patients with covid-19. Diagnostics. 2021;11:1622. 34573964 10.3390/diagnostics11091622 PMC8467337

[32] Smith N, Goncalves P, Charbit B, Grzelak L, Beretta M, Planchais C Distinct systemic and mucosal immune responses during acute SARS-CoV-2 infection. Nature Immunology 2021 22:11 2021; 22: 1428–1439. 10.1038/s41590-021-01028-7 PMC8553615 34471264

[33] Winkley K, Banerjee D, Bradley T, Koseva B, Cheung WA, Selvarangan R Immune cell residency in the nasal mucosa may partially explain respiratory disease severity across the age range. Scientific Reports 2021 11:1 2021; 11: 1–9. 10.1038/s41598-021-95532-3 PMC8342554 34354210

[34] Di Gioia M, Poli V, Tan PJ, Spreafico R, Chu A, Cuenca AG, Epigenetic silencing of interleukin-10 by host-derived oxidized phospholipids supports a lethal inflammatory response to infections. Immunity. 2025;58:2190–e220713. 40680750 10.1016/j.immuni.2025.06.017 PMC12313134

[35] Rojas-Vargas J, Wilcox H, Monari B, Gajer P, Zuanazzi D, Shouldice A The Neovaginal Microbiota, Symptoms, and Local Immune Correlates in Transfeminine Individuals with Penile Inversion Vaginoplasty. bioRxiv 2025;: 2025.03.14.643288. 10.1016/j.celrep.2025.116546 PMC12825010 41352345

[36] Cohen MC, Cohen S. Cytokine function: A study in biologic diversity. Am J Clin Pathol. 1996;105:589–98. 8623768 10.1093/ajcp/105.5.589

[37] Kimura H, Yoshizumi M, Ishii H, Oishi K, Ryo A. Cytokine production and signaling pathways in respiratory virus infection. Front Microbiol. 2013;4:276. 24062733 10.3389/fmicb.2013.00276 PMC3774987

[38] Lozhkov AA, Klotchenko SA, Ramsay ES, Moshkoff HD, Moshkoff DA, Vasin AV, The Key Roles of Interferon Lambda in Human Molecular Defense against Respiratory Viral Infections. Pathogens. 2020;9:989. 33255985 10.3390/pathogens9120989 PMC7760417

[39] Denney L, Ho LP. The role of respiratory epithelium in host defence against influenza virus infection. Biomed J. 2018;41:218. 30348265 10.1016/j.bj.2018.08.004 PMC6197993

[40] Guglani L, Khader SA. Th17 cytokines in mucosal immunity and inflammation. Curr Opin HIV AIDS. 2010;5:120. 20543588 10.1097/COH.0b013e328335c2f6 PMC2892849

[41] Rojas JM, Avia M, Martín V, Sevilla N. IL-10: A multifunctional cytokine in viral infections. J Immunol Res 2017; 2017. 10.1155/2017/6104054. PMC5337865 28316998

[42] Guo-Parke H, Linden D, Mousnier A, Scott IC, Killick H, Borthwick LA, Altered Differentiation and Inflammation Profiles Contribute to Enhanced Innate Responses in Severe COPD Epithelium to Rhinovirus Infection. Front Med (Lausanne). 2022;9. 10.3389/FMED.2022.741989. PMC8916560 35280870

[43] Cheemarla NR, Watkins TA, Mihaylova VT, Wang B, Zhao D, Wang G, Dynamic innate immune response determines susceptibility to SARS-CoV-2 infection and early replication kinetics. J Exp Med. 2021;218. 10.1084/JEM.20210583. PMC8210587 34128960

[44] Kim YM, Shin EC. Type I and III interferon responses in SARS-CoV-2 infection. Exp Mol Med. 2021;53:750–60. 33953323 10.1038/s12276-021-00592-0 PMC8099704

[45] Lin F, ching, Young HA, Interferons. Success in anti-viral immunotherapy. Cytokine Growth Factor Rev. 2014;25:369–76. 25156421 10.1016/j.cytogfr.2014.07.015 PMC4182113

[46] Shi N, Li N, Duan X, Niu H. Interaction between the gut microbiome and mucosal immune system. Mil Med Res. 2017;4. 10.1186/S40779-017-0122-9. 28465831 PMC5408367

[47] Berger A. Science commentary: Th1 and Th2 responses: What are they? Br Med J. 2000;321:424. 10938051 10.1136/bmj.321.7258.424 PMC27457

[48] Prete G, Del. Human Th1 and Th2 lymphocytes: their role in the pathophysiology of atopy. Allergy. 1992;47:450–5. 1485646 10.1111/j.1398-9995.1992.tb00662.x

[49] Kany S, Vollrath JT, Relja B. Cytokines in inflammatory disease. Int J Mol Sci. 2019;20. 10.3390/IJMS20236008. 31795299 PMC6929211

[50] Lam VHK, Ghafoor A, Khan Y, Constable S, Buchanan LB, Zuanazzi D, Protocol for generating and characterizing a nasal epithelial model using imaging with application for respiratory viruses. STAR Protoc. 2025;6:103520. 39772385 10.1016/j.xpro.2024.103520 PMC11760824

[51] Kenney JS, Baker C, Welch MR, Altman LC. Synthesis of interleukin-1α, interleukin-6, and interleukin-8 by cultured human nasal epithelial cells. J Allergy Clin Immunol. 1994;93:1060–7. 8006310 10.1016/s0091-6749(94)70055-9

[52] Blazek K, Eames HL, Weiss M, Byrne AJ, Perocheau D, Pease JE, IFN-λ resolves inflammation via suppression of neutrophil infiltration and IL-1β production. J Exp Med. 2015;212:845–53. 25941255 10.1084/jem.20140995 PMC4451128

[53] Klinkhammer J, Schnepf D, Ye L, Schwaderlapp M, Gad HH, Hartmann R, IFN-λ prevents influenza virus spread from the upper airways to the lungs and limits virus transmission. Elife. 2018;7:e33354. 29651984 10.7554/eLife.33354 PMC5953542

[54] Broggi A, Granucci F, Zanoni I. Type III interferons: Balancing tissue tolerance and resistance to pathogen invasion. J Exp Med. 2020;217. 10.1084/JEM.20190295/132623. PMC7037241 31821443

[55] Mordstein M, Neugebauer E, Ditt V, Jessen B, Rieger T, Falcone V, Lambda Interferon Renders Epithelial Cells of the Respiratory and Gastrointestinal Tracts Resistant to Viral Infections. J Virol. 2010;84:5670–7. 20335250 10.1128/JVI.00272-10 PMC2876583

[56] Salka K, Abutaleb K, Chorvinsky E, Thiruvengadam G, Arroyo M, Gomez JL, IFN stimulates ACE2 expression in pediatric airway epithelial cells. Am J Respir Cell Mol Biol. 2021;64:515–8. 33544656 10.1165/rcmb.2020-0352LE PMC8008803

[57] Su S, Jiang S. A suspicious role of interferon in the pathogenesis of SARS-CoV-2 by enhancing expression of ACE2. Signal Transduction and Targeted Therapy 2020 5:1 2020; 5: 1–2. 10.1038/s41392-020-0185-z PMC7239689 32435059

[58] Ziegler CGK, Allon SJ, Nyquist SK, Mbano IM, Miao VN, Tzouanas CN, SARS-CoV-2 Receptor ACE2 Is an Interferon-Stimulated Gene in Human Airway Epithelial Cells and Is Detected in Specific Cell Subsets across Tissues. Cell. 2020;181:1016. 32413319 10.1016/j.cell.2020.04.035 PMC7252096

[59] Jamilloux Y, Henry T, Belot A, Viel S, Fauter M, El Jammal T, Should we stimulate or suppress immune responses in COVID-19? Cytokine and anti-cytokine interventions. Autoimmun Rev. 2020;19:102567. 32376392 10.1016/j.autrev.2020.102567 PMC7196557

[60] Mordstein M, Kochs G, Dumoutier L, Renauld JC, Paludan SR, Klucher K, Interferon-λ Contributes to Innate Immunity of Mice against Influenza A Virus but Not against Hepatotropic Viruses. PLoS Pathog. 2008;4:e1000151. 18787692 10.1371/journal.ppat.1000151 PMC2522277

[61] Galani IE, Triantafyllia V, Eleminiadou EE, Koltsida O, Stavropoulos A, Manioudaki M, Interferon-λ Mediates Non-redundant Front-Line Antiviral Protection against Influenza Virus Infection without Compromising Host Fitness. Immunity. 2017;46:875–e8906. 28514692 10.1016/j.immuni.2017.04.025

[62] Prokunina-Olsson L, Alphonse N, Dickenson RE, Durbin JE, Glenn JS, Hartmann R, COVID-19 and emerging viral infections: The case for interferon lambda. J Exp Med. 2020;217. 10.1084/JEM.20200653/151664. PMC7155807 32289152

[63] Lazear HM, Schoggins JW, Diamond MS. Shared and Distinct Functions of Type I and Type III Interferons. Immunity. 2019;50:907–23. 30995506 10.1016/j.immuni.2019.03.025 PMC6839410

[64] Feng E, Balint E, Vahedi F, Ashkar AA. Immunoregulatory Functions of Interferons During Genital HSV-2 Infection. Front Immunol. 2021;12:724618. 34484233 10.3389/fimmu.2021.724618 PMC8416247

[65] Lee AJ, Ashkar AA. The dual nature of type I and type II interferons. Front Immunol. 2018;9:403701. 10.3389/fimmu.2018.02061 PMC6141705 30254639

[66] Walker FC, Sridhar PR, Baldridge MT. Differential roles of interferons in innate responses to mucosal viral infections. Trends Immunol. 2021;42:1009. 34629295 10.1016/j.it.2021.09.003 PMC8496891

[67] Chong Z, Karl CE, Halfmann PJ, Kawaoka Y, Winkler ES, Keeler SP, Nasally delivered interferon-λ protects mice against infection by SARS-CoV-2 variants including Omicron. Cell Rep. 2022;39:110799. 35523172 10.1016/j.celrep.2022.110799 PMC9021357

[68] Planet PJ, Parker D, Cohen TS, Smith H, Leon JD, Ryan C, Lambda Interferon Restructures the Nasal Microbiome and Increases Susceptibility to Staphylococcus aureus Superinfection. mBio. 2016;7. 10.1128/MBIO.01939-15. PMC4752601 26861017

[69] Xu W, Larbi A. Immunity and Inflammation: From Jekyll to Hyde. Exp Gerontol. 2018;107:98–101. 29187316 10.1016/j.exger.2017.11.018

[70] Fulop T, Larbi A, Dupuis G, Page A, Le, Frost EH, Cohen AA, Immunosenescence and inflamm-aging as two sides of the same coin: Friends or Foes? Front Immunol. 2018;8:328099. 10.3389/fimmu.2017.01960 PMC5767595 29375577

[71] Brightling C, Berry M, Amrani Y. Targeting TNF-α: A novel therapeutic approach for asthma. J Allergy Clin Immunol. 2008;121:5–10. 18036647 10.1016/j.jaci.2007.10.028 PMC3992375

[72] Al-Ramli W, Préfontaine D, Chouiali F, Martin JG, Olivenstein R, Lemière C, TH17-associated cytokines (IL-17A and IL-17F) in severe asthma. J Allergy Clin Immunol. 2009;123:1185–7. 19361847 10.1016/j.jaci.2009.02.024

[73] Ciprandi G, De Amici M, Murdaca G, Fenoglio D, Ricciardolo F, Marseglia G, Serum interleukin-17 levels are related to clinical severity in allergic rhinitis. Allergy. 2009;64:1375–8. 19226302 10.1111/j.1398-9995.2009.02010.x

[74] Wilson RH, Whitehead GS, Nakano H, Free ME, Kolls JK, Cook DN. Allergic sensitization through the airway primes Th17-dependent neutrophilia and airway hyperresponsiveness. Am J Respir Crit Care Med. 2009;180:720–30. 19661246 10.1164/rccm.200904-0573OC PMC2778149

[75] Ullah MA, Revez JA, Loh Z, Simpson J, Zhang V, Bain L, Allergen-induced IL-6 trans-signaling activates γδ T cells to promote type 2 and type 17 airway inflammation. J Allergy Clin Immunol. 2015;136:1065–73. 25930193 10.1016/j.jaci.2015.02.032

[76] Beenakker KGM, Westendorp RGJ, De Craen AJM, Chen S, Raz Y, Ballieux BEPB, Men Have a Stronger Monocyte-Derived Cytokine Production Response upon Stimulation with the Gram-Negative Stimulus Lipopolysaccharide than Women: A Pooled Analysis Including 15 Study Populations. J Innate Immun. 2020;12:142–53. 31230049 10.1159/000499840 PMC7098282

[77] Butts CL, Shukair SA, Duncan KM, Bowers E, Horn C, Belyavskaya E, Progesterone inhibits mature rat dendritic cells in a receptor-mediated fashion. Int Immunol. 2007;19:287–96. 17289656 10.1093/intimm/dxl145

[78] Klein SL, Flanagan KL. Sex differences in immune responses. Nat Rev Immunol. 2016;16:626–38. 27546235 10.1038/nri.2016.90

[79] Zhang N, Van Zele T, Perez-Novo C, Van Bruaene N, Holtappels G, DeRuyck N, Different types of T-effector cells orchestrate mucosal inflammation in chronic sinus disease. J Allergy Clin Immunol. 2008;122:961–8. 18804271 10.1016/j.jaci.2008.07.008

[80] Diaz-Sanchez D, Rumold R, Gong H. Challenge with environmental tobacco smoke exacerbates allergic airway disease in human beings. J Allergy Clin Immunol. 2006;118:441–6. 16890770 10.1016/j.jaci.2006.04.047

[81] Noah TL, Zhou H, Monaco J, Horvath K, Herbst M, Jaspers I. Tobacco smoke exposure and altered nasal responses to live attenuated infuenza virus. Environ Health Perspect. 2011;119:78–83. 20920950 10.1289/ehp.1002258 PMC3018504

[82] Faden AI, Demediuk P, Panter SS, Vink R. The role of excitatory amino acids and NMDA receptors in traumatic brain injury. Sci (1979). 1989;244:798–800. 10.1126/science.2567056 2567056

[83] Rebuli ME, Glista-Baker E, Hoffman JR, Duffney PF, Robinette C, Speen AM, Electronic-cigarette use alters nasal mucosal immune response to live-attenuated influenza virus: A clinical trial. Am J Respir Cell Mol Biol. 2021;64:126–37. 33095645 10.1165/rcmb.2020-0164OC PMC7781000

[84] Prunas O, Asare EO, Sajewski E, Li Y, Pithawala Z, Weinberger DM, Global estimates of rotavirus vaccine efficacy and effectiveness: a rapid review and meta-regression analysis. EClinicalMedicine. 2025;81. 10.1016/j.eclinm.2025.103122. PMC11925534 40115174

[85] Patriarca PA, Wright PF, John TJ. Factors affecting the immunogenicity of oral poliovirus vaccine in developing countries: Review. Rev Infect Dis. 1991;13:926–39. 1660184 10.1093/clinids/13.5.926

[86] Kim HJ, Jo A, Jeon YJ, An S, Lee KM, Yoon SS, Nasal commensal Staphylococcus epidermidis enhances interferon-λ-dependent immunity against influenza virus. Microbiome. 2019;7. 10.1186/S40168-019-0691-9. 31146794 PMC6542144

[87] Brown RL, Sequeira RP, Clarke TB. The microbiota protects against respiratory infection via GM-CSF signaling. Nature Communications 2017 8:1 2017; 8: 1512-. 10.1038/s41467-017-01803-x PMC5688119 29142211

[88] Bradley KC, Finsterbusch K, Schnepf D, Crotta S, Llorian M, Davidson S, Microbiota-Driven Tonic Interferon Signals in Lung Stromal Cells Protect from Influenza Virus Infection. Cell Rep. 2019;28:245–e2564. 31269444 10.1016/j.celrep.2019.05.105

[89] Kotenko SV, Rivera A, Parker D, Durbin JE. Type III IFNs: Beyond antiviral protection. Semin Immunol. 2019;43. 10.1016/j.smim.2019.101303. PMC7141597 31771761

[90] Mayerhöfer T, Joannidis M, Klein S, Franke A, Margarita S, Ronzoni L The common genetic variant rs1278960 determining expression of Interferon-lambda predicts inflammatory response in critically ill COVID-19 patients. Scientific Reports 2025 15:1 2025; 15: 15802-. 10.1038/s41598-025-91628-2 PMC12056047 40328868

[91] Smith AJP, Humphries SE. Cytokine and cytokine receptor gene polymorphisms and their functionality. Cytokine Growth Factor Rev. 2009;20:43–59. 19038572 10.1016/j.cytogfr.2008.11.006

[92] Dutra WO, Moreira PR, Souza PEA, Gollob KJ, Gomez RS. Implications of cytokine gene polymorphisms on the orchestration of the immune response: Lessons learned from oral diseases. Cytokine Growth Factor Rev. 2009;20:223–32. 19502097 10.1016/j.cytogfr.2009.05.005

[93] Gentile DA, Doyle WJ, Zeevi A, Howe-Adams J, Kapadia S, Trecki J, Cytokine gene polymorphisms moderate illness severity in infants with respiratory syncytial virus infection. Hum Immunol. 2003;64:338–44. 12590978 10.1016/s0198-8859(02)00827-3

[94] Rashu R, Ninkov M, Wardell CM, Benoit JM, Wang NI, Meilleur CE, Targeting the MR1-MAIT cell axis improves vaccine efficacy and affords protection against viral pathogens. PLoS Pathog. 2023;19:e1011485. 37384813 10.1371/journal.ppat.1011485 PMC10337970

[95] Cheng KJ, Zhou ML, Xu YY, Zhou SH. The role of local allergy in the nasal inflammation. European Archives of Oto-Rhino-Laryngology 2017 274:9 2017; 274: 3275–3281. 10.1007/s00405-017-4640-6 28625008

[96] Rondón C, Fernández J, López S, Campo P, Doña I, Torres MJ, Nasal inflammatory mediators and specific IgE production after nasal challenge with grass pollen in local allergic rhinitis. J Allergy Clin Immunol. 2009;124:1005–e10111. 19796796 10.1016/j.jaci.2009.07.018

[97] Chou CK, Winker R, Rebuli ME, Moran T, Rager JE. Respiratory Health Impacts from Natural Disasters and Other Extreme Weather Events: The Role of Environmental Stressors on Asthma and Allergies. Curr Allergy Asthma Rep. 2025;25. 10.1007/S11882-025-01206-9. 40397190 PMC12571042

[98] Singh N, Sharma S. Molecular and Immunological Mechanisms Associated with Diesel Exhaust Exposure. Targets 2025, Vol 3, Page 14 2025; 3: 14.

[99] Shusterman D. The effects of air pollutants and irritants on the upper airway. Proc Am Thorac Soc. 2011;8:101–5. 21364227 10.1513/pats.201003-027RN

[100] Cobos-Uribe C, Dhingra R, Almond MA, Alexis NE, Peden DB, Roach J, Human Sputum Microbiome Composition and Sputum Inflammatory Cell Profiles Are Altered with Controlled Wood Smoke Exposure as a Model for Wildfire Smoke. Am J Respir Crit Care Med. 2025;211:2060–71. 40377699 10.1164/rccm.202407-1493OC PMC12619000

[101] Martin EM, Clapp PW, Rebuli ME, Pawlak EA, Glista-Baker E, Benowitz NL E-cigarette use results in suppression of immune and inflammatory-response genes in nasal epithelial cells similar to cigarette smoke. https://doi.org/101152/ajplung001702016 2016; 311: L135–44. 10.1152/ajplung.00170.2016 PMC4967187 27288488

[102] Rodrigues FMM, Ramos D, Xavier RF, Ito JT, De Souza AP, Fernandes RA, Nasal and systemic inflammatory profile after short term smoking cessation. Respir Med. 2014;108:999–1006. 24863424 10.1016/j.rmed.2014.04.020

[103] Hoagland DA, Møller R, Uhl SA, Oishi K, Frere J, Golynker I, Leveraging the antiviral type I interferon system as a first line of defense against SARS-CoV-2 pathogenicity. Immunity. 2021;54:557–e5705. 33577760 10.1016/j.immuni.2021.01.017 PMC7846242

[104] Major J, Crotta S, Llorian M, McCabe TM, Gad HH, Priestnall SL, Type I and III interferons disrupt lung epithelial repair during recovery from viral infection. Sci (1979). 2020;369:712–7. 10.1126/science.abc2061 PMC7292500 32527928

[105] Mirmozaffari Y, Ly M, Benaim EH, Barron LO, Rebuli ME. Cytokine Sampling in the Nasal Cavity and Paranasal Sinuses. Ear Nose Throat J. 2025. 10.1177/01455613251346570. PMC12777681 40538114

[106] Silva MJA, Ribeiro LR, Gouveia MIM, Marcelino B, dos R, Santos CS dos, Lima KVB Hyperinflammatory Response in COVID-19: A Systematic Review. Viruses 2023, Vol 15, Page 553 2023; 15: 553. 10.3390/v15020553 PMC9962879 36851766

